# Influence of Provider Characteristics on Geriatric Mental Health Workforce Training Needs

**DOI:** 10.1093/geroni/igaa057.3321

**Published:** 2020-12-16

**Authors:** Travis Mitchell, Kristen Sorocco, Mary Wyman, Andrea Vincent, Laura Wray, Lindsey Slaughter

**Affiliations:** 1 University of Oklahoma Health Sciences Center, Oklahoma City, Oklahoma, United States; 2 University of Wisconsin, Madison, Wisconsin, United States; 3 University of Oklahoma, Norman, Oklahoma, United States; 4 Veterans Health Administration, Buffalo, New York, United States; 5 Richmond VAMC, Richmond, Virginia, United States

## Abstract

Approximately half of VA users are 65 years of age or older, with a substantial subset having complex and interacting medical, neurocognitive, and behavioral disorders. The goal of the present study was to assess knowledge and training gaps of VA mental health staff using a Web-based training needs assessment developed specifically for VA personnel. Provider characteristics, confidence in working with older adults with and without cognitive disorder, and geriatric training needs were assessed. VA psychologists, psychiatrists, social workers, nurses, and peer support specialists (N=3313) were invited to participate via email. Respondents were 489 mental health providers, a response rate of 13.8 percent. Respondents reported less confidence in treating and assessing older adults with cognitive disorders then older adults in general. This did not differ by any of the examined provider characteristics, including age, gender, or professional experience. Training need endorsement was high across most of the training categories; however, a few differences according to provider characteristics were noted. Providers under the age of 50 endorsed a greater need for training in psychotherapy with older adults with cognitive disorder (p = .02). Female providers endorsed a greater need for training on providing psychoeducation about cognitive disorder, such as dementia, to older adults and their families (p = .02). In sum, VA MH providers in general reported strong interest in a wide range of geriatric MH training topics, indicating a need for universal geriatric-related staff education. For some topics, endorsing geriatric expertise predicted stronger interest in training.

